# Antimicrobial Resistance Surveillance of Pigs and Chickens in the Lao People’s Democratic Republic, 2018–2021

**DOI:** 10.3390/antibiotics11020177

**Published:** 2022-01-29

**Authors:** Phouth Inthavong, Somphaivanh Chanthavong, Phounsavanh Nammanininh, Phouvong Phommachanh, Watthana Theppangna, Agnes Agunos, Jaap A. Wagenaar, Bounlom Douangngeun, Leo Loth

**Affiliations:** 1 Department of Livestock and Fisheries, Ministry of Agriculture and Forestry, Vientiane 01001, Laos; drphouth@gmail.com (P.I.); wtheppangna@hotmail.com (W.T.); 2 National Animal Health Laboratory, Department of Livestock and Fisheries, Ministry of Agriculture and Forestry, Vientiane 01001, Laos; somphaivanh@yahoo.com (S.C.); noy_phounsavanh@hotmail.com (P.N.); Phouvong.Phommachanh335@outlook.com (P.P.); 3 Food and Agriculture Organization of the United Nations, Regional Office for Asia and the Pacific, Bangkok 10200, Thailand; agnes.agunos@phac-aspc.gc.ca; 4 Department Biomolecular Health Sciences-Infectious Diseases and Immunology, Faculty of Veterinary Medicine, Utrecht University, 3584 CL Utrecht, The Netherlands; j.wagenaar@uu.nl; 5 WHO—Collaborating Center for Campylobacter and Antimicrobial Resistance from a One Health Perspective, OIE Reference Laboratory for Campylobacteriosis, 3584 CL Utrecht, The Netherlands; 6 Food and Agriculture Organization of the United Nations, Country Office for Lao PDR, Vientiane 01001, Laos; Bounlom.Douangngeun@fao.org

**Keywords:** pigs, native chickens, layers, broilers, *Salmonella*, *Escherichia coli*, antimicrobial resistance, multiclass resistance, livestock, surveillance

## Abstract

The use of antimicrobials in the livestock sector has been identified as a driver for the emergence of antimicrobial resistance (AMR), and AMR has become a growing public health and economic threat in the Lao PDR. We conducted surveillance for AMR in five provinces of the Lao PDR, in order to determine the antimicrobial susceptibility of *Escherichia coli* and *Salmonella* spp. isolated from caecal samples from slaughtered pigs at slaughterhouses and from slaughtered chickens at markets during two different time periods: 2018/2019 and 2020/2021. Antimicrobial susceptibility was determined using a panel of 14 antimicrobials using the broth microdilution technique. *E. coli* and *Salmonella* from chickens (62% and 33%, respectively) and pigs (88% and 81%, respectively) exhibited resistance to ≥3 classes of antimicrobials. Of important public health concern was the detection of *Salmonella* resistant to cefotaxime/ceftazidime, ciprofloxacin, and colistin, deemed as critically important antimicrobials in human medicine. This study aimed to evaluate a national sampling strategy at slaughterhouses and wet markets, and to pilot the laboratory methodologies for bacterial recovery and AMR testing. Experiences from this study will inform capacity development for a national AMR surveillance program, and these early data could serve as reference points for monitoring the impact of the Lao PDR’s national action plan to contain AMR.

## 1. Introduction

Large quantities of a variety of classes of antimicrobials are used in the livestock industry in Southeast Asia [1,2]. Unregulated and irresponsible use of antimicrobials in the animal production sector can result in antimicrobial-resistant (AMR) bacteria [3,4]. Food safety and public health threats arising from the use of antimicrobials in food animals—including antimicrobial-resistant bacteria/genetic elements and antimicrobial residues—can spread through direct contact with animals, animal excrement, wastewater, vegetables, and animal byproducts [5,6,7]. Inappropriate use of antimicrobials in people contributes to the issue of AMR, which is concerning because infections with antimicrobial-resistant organisms may lead to therapeutic failure and, thus, impact the burden of illness in a country. In animal production settings, this could potentially decrease production performance parameters, leading to substantial economic losses [8].

**Figure 1:**
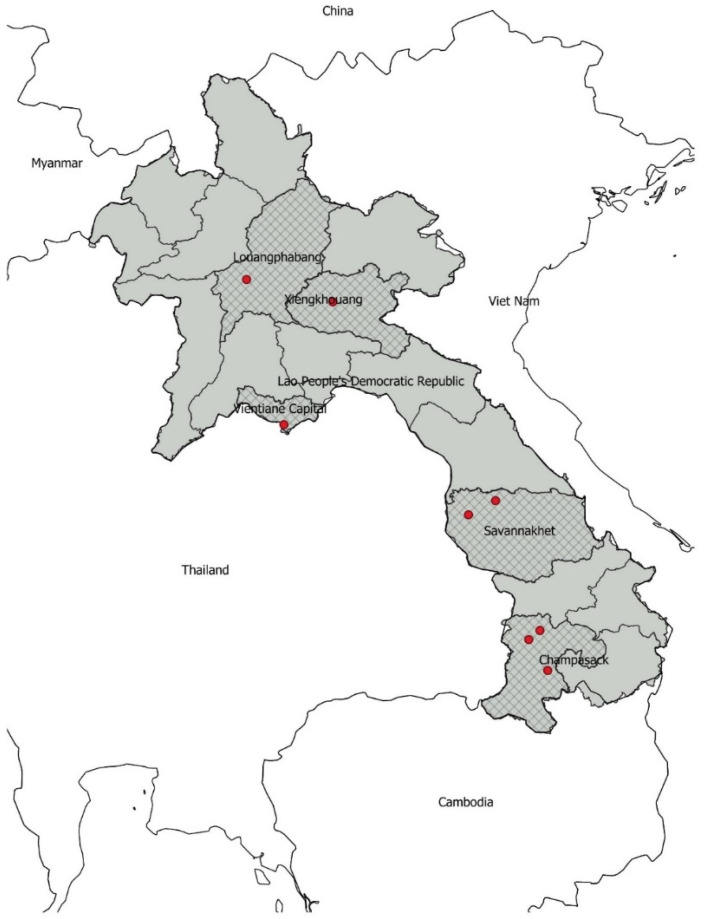
The Lao People’s Democratic Republic (grey) and its neighboring countries; also shown: the location of the 8 districts (red dots) in 5 provinces where the sampling was conducted.

In the Southeast Asia region, in countries bordering the Lao PDR (Figure 1), indicator and foodborne bacterial organisms revealed high to extremely high levels of resistance to antimicrobials, suggesting that antimicrobials commonly used for the therapy of bacterial diseases may no longer be efficacious. For instance, *Escherichia coli* isolated from pig and chicken farms in Vietnam [9] and Cambodia [3] showed up to 97% resistance to ampicillin, 73.3% resistance to ciprofloxacin, 42.2% resistance to gentamicin, and 24.4% resistance to colistin. High AMR prevalence was measured in *Salmonella enterica* isolated from broiler chickens, pigs, and meat products in Thailand and Cambodia [10]. Resistance to several antimicrobials was also detected in *Salmonella* spp. [11] and *E. coli* [12] in Myanmar. Studies in border provinces in the Lao PDR reported colistin resistance in *E. coli* from pigs [13] and *E. coli* from humans and pigs [7]. As all of these countries extensively trade animals, animal products, and antimicrobials, AMR surveillance in food animals and their products in districts/provinces of the Lao PDR contiguous to these bordering countries, and where these products are distributed, are primary considerations for designing a national AMR surveillance program.

In 2019, the National Strategic Plan on Antimicrobial Resistance (NSP-AMR) in the Lao PDR, 2019–2023, was developed by the Ministry of Health and the Ministry of Agriculture and Forestry, with technical and financial support from the [14] World Health Organization (WHO), the Food and Agriculture Organization of the United Nations (FAO), and the World Organization for Animal Health (OIE) [15]. The NSP-AMR aligns with the Global Action Plan [16], the FAO Action Plan on AMR 2021–2025 [17] and its predecessor [18], and the OIE AMR strategy [19]. This strategic plan on AMR describes the vision to reduce AMR-related human and animal morbidity, mortality, and economic impact. The strategic plan encourages a multisector One Health approach to tackling AMR, involving local and national stakeholders, and demonstrates the commitment of the Lao PDR in the global fight against AMR.

One of the strategic objectives of the NSP-AMR is to strengthen the AMR surveillance system. As previously described, there are very few specific national studies on AMR in livestock in the Lao PDR. These data gaps hampered our assessment of the “state of science” of AMR in the country. Thus, in collaboration with the FAO, the Department of Livestock of Fisheries of the Ministry of Agriculture and Fisheries conducted a pilot surveillance project in chickens and pigs to inform the development of sampling and laboratory methodologies for an ongoing national AMR surveillance program, and to generate baseline bacterial recovery and AMR data that could be used by the country as reference points for monitoring the progress of the NSP-AMR. This surveillance was designed to determine the levels of resistance to antimicrobials in *E. coli*—an indicator organism broadly used in AMR surveillance—and *Salmonella* spp., an important foodborne zoonotic pathogen in the Lao PDR [20]. This pilot project sampled chickens and pigs—the two animal species deemed as essential to food security in the Lao PDR.

## 2. Materials and Methods

### 2.1. Sampling Design

The surveillance was conducted in eight districts in five provinces in the Lao PDR (Figure 1). The target population was animals closest to the consumers, such as pigs at slaughter points and chickens sold at wet markets. The surveillance was conducted in pigs with a first sampling in 2018 and 2019 in three provinces: Vientiane Capital, Savannakhet, and Champasak. The second round of sampling in pigs was conducted in 2020 and 2021, in two additional provinces: Luang Prabang and Xiengkhouang. In poultry, the sampling was performed in 2020 and 2021 in all five mentioned provinces (Vientiane Capital, Savannakhet, Champasak, Luang Prabang, and Xiengkhouang). Three types of poultry were sampled, depending on their availability in the different provinces (native breeds, commercial broilers, and layers).

### 2.2. Sample Collection

With the use of sterilized dissecting tools and packaging materials (labelled per sample), provincial laboratory staff aseptically collected caecal samples of slaughtered healthy pigs in the slaughterhouse and caecal samples of slaughtered healthy poultry at the slaughter points at live bird markets. In chickens, the caecum was collected by placing a ligature where the small and large intestine join and separated by scissors. In pigs, caecal content was aseptically collected by disinfecting the caecal wall, opening the intestinal wall (incision using sterile scissors as above), and collecting caecal contents directly in a sterile sampling cup. Per animal sampled, the caeca/caecal contents were placed in a sterile plastic bag and stored at 4 °C. For both sample collection rounds 1 and 2, every week, an average of 10 caecal samples were collected from pigs, broilers, layers, and native chickens in each province. Provincial laboratory staff were asked to ensure that samples were epidemiologically independent (not from animals from the same farms/flocks/herds). For the total sample size, the European Food Safety Authority’s (EFSA) target sample size of 170 isolates tested for each species [21] was used. The total sample size of 350 for each species collected each round and for each province was assumed to yield at least 170 *Salmonella* spp. isolates if the prevalence of *Salmonella* spp. was 50% in pigs and chickens [22].

### 2.3. Sample Processing and Bacterial Recovery

The National Animal Health Laboratory (NAHL) in Vientiane, which is an assigned laboratory for AMR surveillance, was assessed for technical capacity and biological material management before conducting the first round of the surveillance using the FAO Assessment Tool for Laboratory and AMR Surveillance Systems (ATLASS) in April 2017 [23]. All laboratory tests were performed at the NAHL based on protocols, following the FAO’s regional antimicrobial resistance surveillance and monitoring guidelines [24]. After field sample collection, the caecal samples from pigs and chickens were submitted to the NAHL for processing and the recovery of *Salmonella* spp. and *E. coli*. In brief, caeca were opened aseptically, and 25 g of caecal content was enriched in 250 mL of buffered peptone water and kept in the incubator at 35–39 °C for 18–24 h. Detection and identification of *Salmonella* spp. and *E. coli* were conducted as described in the FAO protocol [24]. All *E. coli* or *Salmonella* spp. isolates were stored at the laboratory and preserved in appropriate conditions before conducting the antimicrobial susceptibility testing. Isolates to be tested and quality control strains (*E. coli* ATCC 25922) were revived by using a nutrient agar medium. *Salmonella* spp. isolates were not further characterized (no serogrouping and no serotyping) for this study.

### 2.4. Antimicrobial Susceptibility Testing (AST)

The susceptibility of *E. coli* and *Salmonella* spp. isolates was determined using the commercial *Sensititre*^TM^ Minimum Inhibitory Concentration (MIC) EUVSEC plates (Thermo Fisher Scientific, Waltham, MA, USA) [25]. The EUVSEC comprised 14 antimicrobials belonging to 8 antimicrobial classes that have significance to public health, including ampicillin, azithromycin, cefotaxime, ceftazidime, chloramphenicol, ciprofloxacin, colistin, gentamicin, meropenem, nalidixic acid, sulfamethoxazole, tetracycline, tigecycline, and trimethoprim. The *Sensititre*^TM^ OptiRead Automated Fluorometric Plate-Reading System (Thermo Fisher Scientific) and SWIN software (Thermo Fisher Scientific) were used for reading the results [25]. Additional quality control test recommendations by the manufacturer [25] and the FAO’s regional recommendations [22] were followed. *E. coli* and *Salmonella* spp. isolates were classified as resistant using the European Committee on Antimicrobial Susceptibility Testing (www.EUCAST.org, accessed 24 June 2021) clinical breakpoints (CBPs) for the following antimicrobials: ampicillin, cefotaxime, ceftazidime, chloramphenicol, ciprofloxacin, colistin, gentamicin, meropenem, tigecycline, and trimethoprim. Where no EUCAST breakpoints were available, in the interim, the Clinical Laboratory Standard Institute (CLSI) [26] breakpoints were used, as with other AMR surveillance systems [27,28] (i.e., for azithromycin, nalidixic acid, sulfamethoxazole, and tetracycline; please refer to the Supplementary Materials, Table S1, for the CBPs used for these antimicrobials).

### 2.5. Data Entry and Storage

Sample information and basic demographics (e.g., geographical location, production type) were entered in Microsoft Excel (Microsoft Office Professional Plus 2016, Microsoft, Redmond, WA, USA) spreadsheets and linked with the MIC data generated by the SWIN software. The animal information (i.e., slaughterhouse and live bird market sources) was assigned with unique codes, and no personal identifiers of the farmer(s) or slaughterhouse operator(s) were collected.

### 2.6. Data Analysis and Interpretation

#### 2.6.1. Validation and Data Preparation

Prior to analysis, data were validated and checked for errors (e.g., duplicates, data entry errors, MIC values/ranges, and missing information). Analyses were conducted using SAS Version 9.4 (SAS, Cary, NC, USA), Stata/SE V16.1 (StataCorp, College Station, TX, USA) and Microsoft Excel (Office Professional 2016, Microsoft).

#### 2.6.2. Descriptive Statistics of MIC and Binary Resistance Information

For each animal species–antimicrobial combination, raw MIC data were tabulated in frequency tables and plotted in Microsoft Excel graphs for visualization. Descriptive statistics were obtained using Stata/SE V16.1 (e.g., percentiles, range), and the median values were marked within the Excel MIC distribution plots for each antimicrobial. For the analysis of the mean resistance and 95% confidence intervals (95% CI) for each antimicrobial, the MIC data were dichotomized into non-resistant or resistant using the EUCAST (or CLSI for the 4 antimicrobials) CBPs described in the Supplementary Materials, Table S1. Throughout this paper, the levels of resistance are described following those of the EFSA, as follows: “rare”: <0.1%, “very low”: 0.1–1.0%; “low”: >1–10.0%; “moderate”: >10.0–20.0%; “high”: >20.0–50.0%; “very high”: >50.0–70.0%; “extremely high”: >70.0% [29].

Additional AMR outcomes such as no resistance and multiclass resistance (resistances to 1–8 classes) were determined, and the distribution of isolates was compared between species. For the purposes of this paper, no resistance refers to isolates that showed susceptibility to the 14 antimicrobials included in the EUVSEC panel, while ≥3 multiclass-resistant isolates refers to those isolates that exhibited resistance to antimicrobials belonging to at least 3 antimicrobial classes [30]. Resistance phenotypes (class) in chickens and pigs were summarized.

#### 2.6.3. Comparison between Chickens and Pigs, and Subanalysis of Chicken Types and Sampling Rounds in Pigs

Differences in resistance outcomes between chickens and pigs were assessed using logistic regression analysis (LOGISTIC procedure in Stata/SE V16.1). For each antimicrobial and other AMR outcomes (no resistance and multiclass resistances), models were built using the binary data (yes/no) as the outcome variable and the species as the categorical independent variable, and adjusted for clustering at the province level. The same modelling approach was used for evaluating the differences in resistance between chicken breeds/production types (independent categorical variable: layer, broiler, native chickens) and differences in resistance between sampling rounds/geographical locations in pigs. Effect estimates (odds ratios (OR), 95% CIs, and *p*-values) were noted, and a *p*-value of 0.05 was considered significant. An OR > 1 or <1 indicates that the probability of resistance increases (greater) or decreases (lower), respectively, between species, between chicken breeds/production profiles, and between pig sample collection rounds/geographical locations.

## 3. Results

### 3.1. Bacterial Isolation

Between 2018 and 2021, a total of 3638 samples from five provinces were tested (Table 1). The overall *E. coli* isolation rate was 87% (3150/3638), while the rate was 58% (2098/3638) for *Salmonella.* There was significantly (*p* < 0.001) more *E. coli* isolated from chickens (90%, 2236/2479) than from pigs (79%, 914/1159), while the *Salmonella* isolation rate was significantly higher in pigs (61%, 711/1159) compared to chickens (56%, 1387/2479) (*p* = 0.002). The isolation rates for *E. coli* (771/825, 744/829, 721/825; *p* = 0.086) were not significantly different when comparing the three poultry breeds (native, broiler, and layer). The isolation rates for *Salmonella* (36%, 301/825, 62%, 511/829, 70%, 575/825; *p* < 0.001) were significantly lower in native birds, compared to broiler and layer chickens. The *E. coli* and *Salmonella* tested for AMR were systematically selected (for *E. coli*, approximately every other isolate in the archive; for *Salmonella*, 66% (two out of three) of the isolates in the archive).

### 3.2. Antimicrobial Resistance in Escherichia coli Isolates

#### 3.2.1. Minimum Inhibitory Concentration (MIC) Distribution

**Figure 2:**
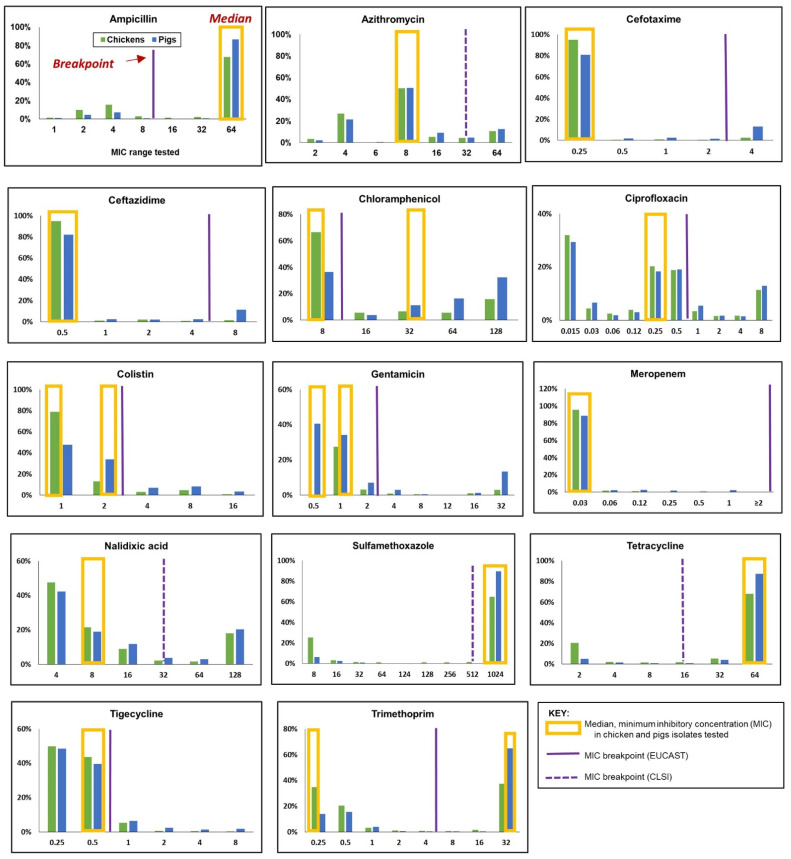
Distribution of minimum inhibitory concentration (MIC) in a panel of 14 antimicrobials, showing the relative locations of the clinical breakpoints and the median MIC values in *Escherichia coli* isolated from chickens (*n* = 1110 isolates) and pigs (*n* = 754).

Figure 2 summarizes the MIC distribution of the 14 antimicrobials included in the EUVSEC panel, along with the relative positions of the CBPs against the median MIC values for chickens and pigs. The distribution of MICs varied depending on the animal species and the antimicrobial. Median MICs in 10 antimicrobials were comparable in chickens and pigs. For colistin and gentamicin, the median MICs were higher in pigs compared to chickens. The median MICs for the following antimicrobials were lower than the EUCAST CBPs: cefotaxime, ceftazidime, ciprofloxacin, colistin, gentamicin, meropenem, and tigecycline. In four antimicrobials, the CLSI CBPs were used in the absence of EUCAST CBPs to characterize the resistance levels for chickens and pigs in this study. Specifically, the median MICs detected for sulfamethoxazole and tetracycline were one dilution (1024 µg/mL) and two dilutions (64 µg/mL) above the CBPs, respectively. On the other hand, the median MICs recorded for azithromycin and nalidixic acid were two dilutions (8 µg/mL) below the CLSI CBPs. The median MICs for antimicrobials categorized as highest priority-critically important antimicrobials (HPCIAs) by the WHO [31] (e.g., cefotaxime (0.25 µg/mL), ceftazidime (0.5 µg/mL) and meropenem (0.01 µg/mL)) corresponded with the lowest dilution ranges for the antimicrobials.

#### 3.2.2. Resistance to Single (Homologous) Antimicrobials

**Figure 3:**
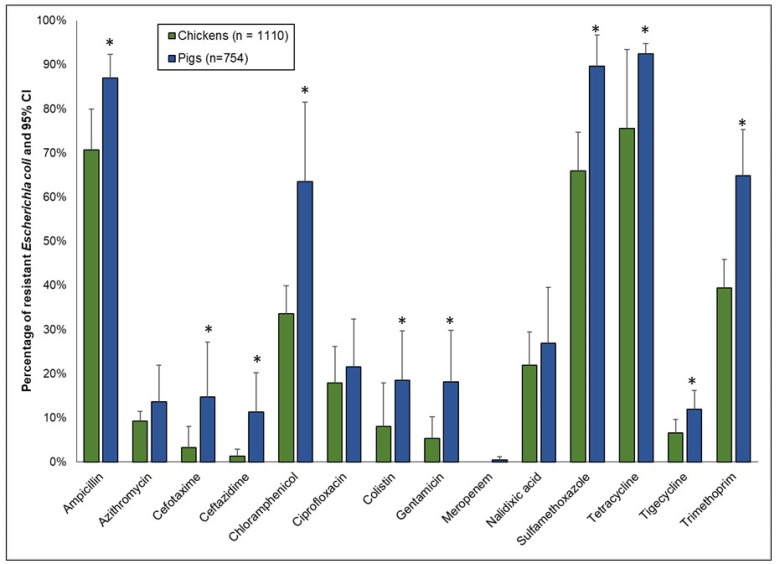
Percentages of antimicrobial resistance in *Escherichia coli* from chickens (*n* = 1110; depicted by the green bars) and pigs (*n* = 754; depicted by the blue bars), collected from five provinces in the Lao PDR, 2018–2021. High 95% confidence limits are shown. For each antimicrobial and animal species combination, the percentage of resistance was adjusted for clustering at the province level. Asterisks represent significant differences (*p* ≤ 0.05) for a given antimicrobial between chickens and pigs.

Figure 3 shows the resistance in *E. coli* isolates from poultry (combined data from layers, broilers, and native chickens) and pigs (combined rounds 1 and 2). Variations in the percentage of resistance were observed between pigs and chicken isolates, depending on the antimicrobial. Noteworthy was the significantly higher resistance to the WHO’s HPCIAs in pigs compared to chickens, including cefotaxime (pigs: 15% vs. chickens: 3%, OR 5.15, *p* < 0.0001), ceftazidime (pigs: 11% vs. chickens: 1%, OR 9.27, *p* < 0.0001). And colistin (pigs: 18% vs. chickens: 8%, OR 2.6, *p* < 0.0001). Relatively moderate resistance to ciprofloxacin was detected, but the difference between the species was not statistically significant (pigs: 21% vs. chickens: 18%). Very low-level resistance to meropenem (<1%) was detected in pig isolates, but not in chickens. For the remaining antimicrobials, moderate-to-high resistances were detected that were significantly higher in pigs compared to chickens: ampicillin (extremely high), chloramphenicol (very high), gentamicin (moderate), sulfamethoxazole (extremely high), tetracycline (extremely high), and trimethoprim (very high).

##### Comparison between Different Chicken Production Types

Variations in the percentages of resistance to antimicrobials in the different poultry types (broilers, layers, and native chickens) were observed (Supplementary Materials, Figure S1). In most cases, broilers had the highest percentage of resistance, but these levels were not statistically significant compared to layers and native chickens. Noteworthy was the lower resistance to the WHO’s HPCIAs, such as ciprofloxacin and colistin, in native chickens.

##### Comparison between Two Time Periods/Geographical Regions of the Lao PDR in Pig Isolates

Resistances to the WHO’s HPCIAs, such as ciprofloxacin and colistin, were not statistically significant between the two rounds of sample collection, but lower resistance during round 2 was detected for the following HPCIAs: cefotaxime (2nd round: 10% vs. 1st round: 16%, OR 0.23, *p* < 0.0001), ceftazidime (2nd round: 7% vs. 1st round: 13%, OR 0.15, *p* < 0.0001) (Supplementary Materials, Figure S2). Additionally, lower resistance during round 2 was detected for other antimicrobials, including gentamicin (2nd round: 13% vs. 1st round: 20%, *p* < 0.0001) and sulfamethoxazole (2nd round: 92% vs. 1st round: 92%, OR 0.19, *p* < 0.0001). Moderate to extremely high resistances to other antimicrobials in the panel were detected (Supplementary Materials, Figure S2), which were significantly higher during the 2nd period compared to the 1st period: ampicillin (extremely high), chloramphenicol (very high), tetracycline (extremely high), and tigecycline (moderate).

#### 3.2.3. Multidrug and Multiclass Resistance

**Figure 4:**
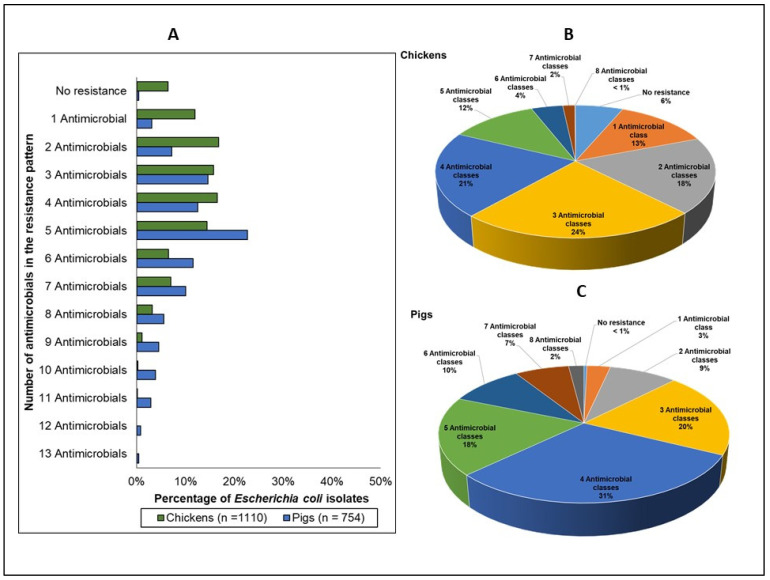
Distribution of the resistance phenotypes (number of individual antimicrobials in the pattern) in *Escherichia coli* from chickens (depicted by the green bars; *n* = 1110) and in pigs (depicted by the blue bars; *n* = 754) (**A**), and the distribution of resistant phenotypes (number of classes in the pattern) in chickens (**B**) and pigs (**C**). Please refer to the Supplementary Materials, Table S2A,B, for the specific antimicrobial resistance phenotypes.

The distribution of *E. coli* resistance phenotypes is shown in Figure 4A. Isolates that exhibited no resistance to any of the 14 antimicrobials in the EUVSEC panel were less common in pigs (referent) (<1%) compared to chickens (6%) (OR 0.06, *p* = 0.003). The maximum number of antimicrobials to which an isolate was resistant was 11 antimicrobials (2 isolates) in chickens and 13 antimicrobials (3 isolates) in pigs.

When individual antimicrobial resistances were aggregated by antimicrobial class (Figure 4B,C), the relative distribution of the isolates varied between chickens and pigs (Table 2). Total isolates that exhibited resistance to antimicrobials belonging to three or more classes of antimicrobials (≥3 multiclass-resistant) significantly differed between the two species (pigs: 88% vs. chickens: 62%, OR 4.29, *p* = 0.001). There were 77 unique multiclass resistance phenotypes identified in chickens (Supplementary Materials, Table S2A), and the top two most frequently occurring phenotypes were a resistance pattern comprising beta-lactams, folate pathway inhibitors, and tetracyclines (155 isolates, 15%), followed by a resistance pattern containing the same three antimicrobial classes above, plus phenicols (131 isolates, 13%). In pigs, there were 67 different multiclass resistance phenotypes (Supplementary Materials, Table S2B), and the most frequently occurring phenotypes were a resistance pattern comprising beta-lactam antimicrobials, folate pathway inhibitors, tetracyclines, and phenicols (174 isolates, 23%), followed by a resistance pattern comprising beta lactams, folate pathway inhibitors, and tetracyclines (124 isolates, 17%).

### 3.3. Antimicrobial Resistance in Salmonella Isolates

#### 3.3.1. MIC Distribution

**Figure 5:**
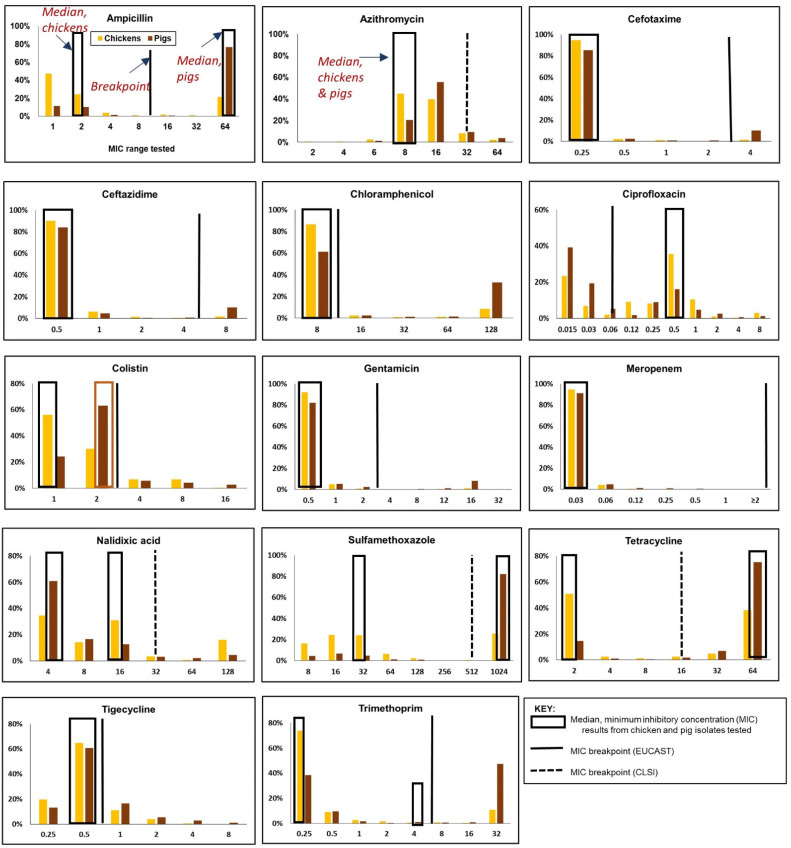
Distribution of minimum inhibitory concentrations (MICs) in a panel of 14 antimicrobials, showing the relative locations of the clinical breakpoints and the median MIC values in *Salmonella* spp. isolated from chickens (*n* = 698 isolates) and pigs (*n* = 673).

Figure 5 summarizes the MIC distribution of the 14 antimicrobials included in the EUVSEC panel and the relative positions of the CBPs against the median MIC values for chickens and pigs. As with *E. coli*, the distribution of MICs varied depending on the species and the antimicrobial. Median MICs in eight antimicrobials were comparable in chickens and pigs. The median MICs for the following antimicrobials were lower than the EUCAST CBPs: cefotaxime, ceftazidime, chloramphenicol, colistin, gentamicin, meropenem, tigecycline, and trimethoprim—in both chicken and pig isolates. For ciprofloxacin, median MICs in chickens and pigs were three dilutions above the CBPs. As with *E. coli*, CLSI CBPs were used in the absence of EUCAST CBPs to characterize the resistance in this study. Specifically, median MICs were below the CLSI CBPs (e.g., azithromycin, nalidixic acid) in both species, but substantially varied for sulfamethoxazole (chickens: 32 µg/mL vs. pigs: 1024) and tetracycline (chickens: 2 µg/mL vs. pigs: 64 µg/mL). As with *E. coli*, the median MICs for the HPCIAs cefotaxime, ceftazidime, and meropenem corresponded with the lowest dilution ranges for these antimicrobials.

#### 3.3.2. Resistance to Single (Homologous) Antimicrobials

**Figure 6:**
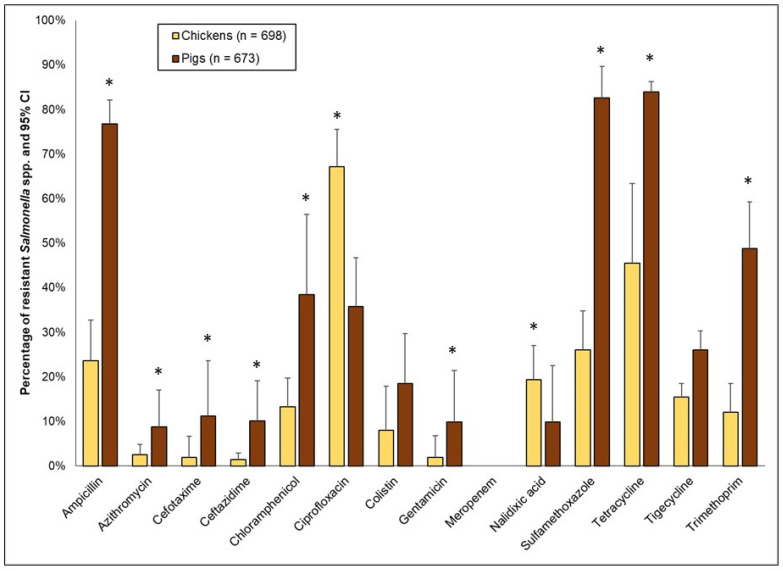
Percentages of antimicrobial resistance in *Salmonella* spp. from chickens (*n* = 698 isolates; depicted by the yellow bars) and pigs (*n* = 673; depicted by the brown bars), collected from five provinces in the Lao PDR, 2018–2021. High 95% confidence limits are shown. For each antimicrobial and species combination, the percentage of resistance was adjusted for clustering at the province level. Asterisks represent significant differences (*p* ≤ 0.05), between chickens and pigs.

Figure 6 shows the percentages of resistance to the 14 antimicrobials in *Salmonella* spp. isolated from poultry (combined; native, broiler, and layer birds) and pigs (combined rounds 1 and 2). As with *E. coli*, variations in the percentage of resistance were observed between pig and chicken isolates, depending on the antimicrobial. Low-to-moderate levels of resistance were noted for the 3rd-generation cephalosporins, and were significantly higher in pigs compared to chickens—cefotaxime (pigs: 11% vs. chickens: 2%, OR 6.55, *p* < 0.0001) and ceftazidime (pigs: 10% vs. chickens: 1%, OR 7.55, *p* < 0.0001). However, the opposite was noted for ciprofloxacin, where resistance in pigs was significantly lower compared to chickens (pigs: 36% vs. chickens: 67%, OR 0.27, *p* = 0.005). These findings mirrored the resistance to nalidixic acid (pigs: 10% vs. chickens: 19%, OR 0.45, *p* = 0.016). High to extremely high levels of resistance (Figure 6) were detected in other antimicrobials, but were significantly higher in pigs compared to chickens—ampicillin (extremely high), chloramphenicol (high), sulfamethoxazole (extremely high), tetracycline (extremely high), and trimethoprim (very high). Colistin resistance was relatively higher in pigs (18%) than in chickens (8%), but the difference was not statistically significant. Unlike in *E. coli*, no resistance was detected for meropenem in *Salmonella* spp. isolates.

##### Comparison between Different Chicken Production Types

Variations in the percentages of resistance to antimicrobials in the different poultry types (broilers, layers, and native chickens) were observed (Supplementary Materials, Figure S3). Notably, for colistin, resistance in layers (referent breed) (20%) was significantly higher compared to broilers (10%, OR 0.45, *p* = 0.013) and native chickens (10%, OR 0.45, *p* = 0.023). As for the quinolone antimicrobials, low-to-moderate levels of ciprofloxacin resistance were detected in the three breeds (no significant differences), but significant variations were observed for nalidixic acid, where layers (27%) were significantly higher compared to broilers (17%, OR 0.30, *p* < 0.0001) and native chickens (10%, OR 0.30, *p* = 0.0001). In most cases (except azithromycin and sulfamethoxazole), resistances in native chickens were relatively lower.

##### Comparison between Two Time Periods/Geographical Regions of the Lao PDR in Pig Isolates

As with *E. coli*, differences in resistance to ciprofloxacin, colistin, cefotaxime, and ceftazidime between the two rounds of sample collection were not statistically significant. However, for certain antimicrobials, compared to the first round, significantly lower levels were detected during the second round—chloramphenicol (2nd round: 34% vs. 1st round: 41%; OR 0.33, *p* = 0.002), sulfamethoxazole (2nd round: 66% vs. 1st round: 93%, OR 0.04, *p* < 0.0001), tetracycline (2nd round: 82% vs. 1st round: 85%, OR 0.21, *p* = 0.007), and trimethoprim (2nd round: 46% vs. 1st round: 40%, OR 0.27, *p* = 0.026) (Supplementary Materials, Figure S4).

#### 3.3.3. Multidrug and Multiclass Resistance

**Figure 7:**
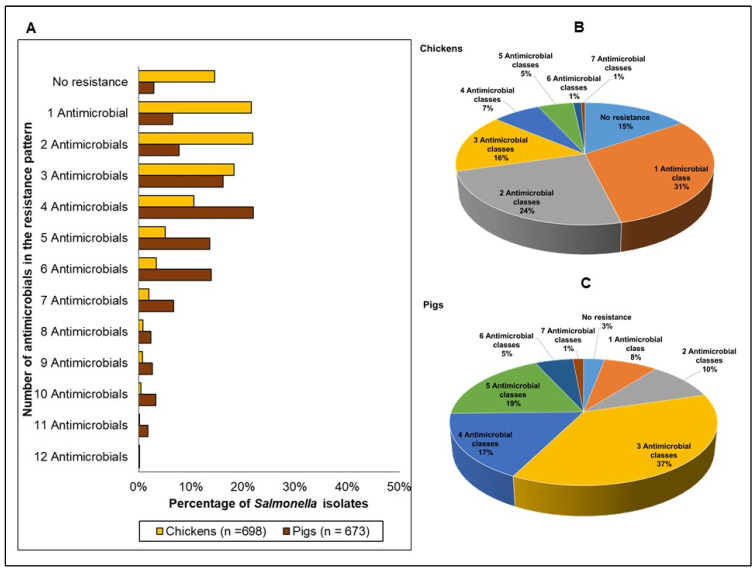
Distribution of the resistance phenotypes (number of individual antimicrobials in the pattern) in *Salmonella* spp. from chickens (depicted by the yellow bars; *n* = 698) and in pigs (depicted by the brown bars; *n* = 675) (**A**), and the distribution of resistant phenotypes (number of classes in the pattern) in chickens (**B**) and pigs (**C**). Please refer to the Supplementary Materials, Table S3A,B, for the specific antimicrobial resistance phenotypes.

The distribution of *Salmonella* resistance phenotypes is shown in Figure 7A. As with *E. coli*, the percentage of isolates that exhibited no resistance to any of the 14 antimicrobials in the EUVSEC panel was lower in pigs (referent) (<3%) compared to chickens (15%) (OR 0.18, *p* < 0.0001). The maximum number of antimicrobials to which an isolate was resistant to was 12 antimicrobials (1 isolate) in both chickens and pigs.

When individual antimicrobial resistances were aggregated by antimicrobial class (Figure 7B,C), the relative distribution of the isolates substantially varied between chickens and pigs in most multiclass resistance categories (Table 3). Total isolates that exhibited resistance to antimicrobials belonging to ≥3 classes significantly differed between the two species (pigs: 81% vs. chickens: 33%, OR 8.53, *p* < 0.0001). There were 64 unique multiclass resistance phenotypes identified in chickens (Supplementary Materials, Table S3A), and the most frequently occurring phenotypes were resistance patterns comprising quinolones (120 isolates, 20%), followed by the pattern comprising folate pathway inhibitors, quinolones, and tetracyclines (52 isolates, 9%). In pigs, there were 65 different multiclass resistance phenotypes (Supplementary Materials, Table S3B), and the top 2 most frequently occurring phenotypes were the resistance pattern comprising beta-lactams, folate synthesis inhibitors, and tetracyclines (174 isolates, 27%), followed by the resistance pattern comprising beta lactams, folate synthesis inhibitors, quinolones, phenicols, and tetracyclines (78 isolates, 12%).

## 4. Discussion

This study explored a national sampling frame from a network of government-monitored slaughterhouses and wet markets in prioritized districts/provinces of the Lao PDR, where the highest concentrations of pig and poultry production in the country and provincial laboratories are conveniently located. Sampling in these provinces ensured that the average chicken or pig farms in the Lao PDR were captured. This study also assessed the laboratory capacity for AMR surveillance in food animals. To the best of our knowledge, this is the first evidence that food animals slaughtered and sold for human consumption in the Lao PDR are frequently contaminated with antimicrobial-resistant bacteria. Of important public health concern is the detection of *Salmonella* spp. resistant to the WHO’s HPCIAs (third-generation cephalosporins, quinolones, and polymyxins). Additionally, detection of *E. coli* isolates resistant to the same classes of antimicrobials is concerning, because these could serve as a reservoir of resistant genes in the environment and in animal populations, posing a food safety and public health threat. In particular, *E. coli* and *Salmonella* isolates from both animal species exhibited resistance to colistin, known as a last resort drug and the medicine of choice for the treatment of serious multidrug-resistant *Enterobacteriaceae* and *Pseudomonas aeruginosa* infections in humans. Colistin resistance was also contained in numerous resistance phenotypes in *E. coli* and *Salmonella* spp. in this study, and warrants closer monitoring in both animal and human populations in the Lao PDR. The detection of isolates with resistance to meropenem—a carbapenem—was very rare. With the exception of ciprofloxacin, percentages of resistance to homologous antimicrobials and ≥multiclass resistance were higher in pigs compared to chickens, necessitating an urgent call for stewardship in the pig sector.

Our data indicated variations in resistance to the WHO’s HPCIAs—such as the 3rd-generation cephalosporins, quinolones, and polymyxins—between pigs and chickens. In most cases, higher levels were observed in pigs compared to chickens, except for the quinolone antimicrobial, ciprofloxacin. The percentages of multiclass-resistant *E. coli* and *Salmonella* were higher in pigs. Furthermore, chickens were contaminated with isolates with multiclass-resistant phenotypes distinct from those found in pigs, signifying potential variations in antimicrobial options for treating specific bacterial infections. However, detailed information on AMU (deemed as the main driver of AMR) and potential risk factors (e.g., biosecurity and farm-level practices) in these species are required in order to better understand variations in AMR and the epidemiology of AMR in the Lao PDR’s food animal sector. In a recent study, AMU was identified as a major knowledge gap in understanding the current status of AMR in the Lao PDR [32]. In another study, antimicrobials belonging to beta-lactam penicillins and fluoroquinolones were reportedly used by pig farmers more frequently than chicken farmers who participated in the research [32], which could partially explain our results (higher levels of ampicillin, cefotaxime, and ceftazidime antimicrobials). Moreover, resistance to older antimicrobial classes (e.g., phenicols, sulfonamides, and tetracyclines) was common in both species, but was observed to be higher in pigs. Low-level resistance to azithromycin—a semi-synthetic macrolide—was detected, which was more pronounced in *E. coli* and *Salmonella* from pigs than from chickens. Other surveillance systems, such as the US NARMS [33], reported occasional detection of azithromycin-resistant *Salmonella* isolates from food animal species, whereas the EFSA reported resistance levels that varied from rare to moderate depending on the country [34]. Without detailed AMU information (i.e., the use of natural macrolides such as erythromycin) and characterization of macrolide resistance determinants from the isolates, it is unclear how resistance to azithromycin emerged in the Lao PDR’s animal populations. The detection of resistance to chloramphenicol in *E. coli* and *Salmonella* spp. is a phenomenon that has been observed in countries where chloramphenicol has not been used for decades. Although this is a finding commonly reported by several surveillance systems across the world [29,33,34,35,36,37], it is important to investigate whether chloramphenicol has been completely banned from animal production, according to the FAO’s plea to all countries to discontinue the use of chloramphenicol in animal production [38]. The roles of other antimicrobials belonging to the phenicols class of antimicrobials (e.g., thiamphenicol) [32] also need to be investigated. These findings suggest that AMU surveillance is equally important, and could complement AMR surveillance in the pursuit of understanding the emergence and spread of AMR organisms within animal populations in the Lao PDR. Surveillance of AMR in clinical pathogens and syndromic disease investigations could be of value for understanding the main drivers of AMU. As surveillance capacity improves, molecular work for characterizing the genetic determinants in archived (including the isolates from this study) and future isolate collections could improve our understanding on how resistance (including co-selection and cross-resistance mechanisms) and multidrug resistance phenotypes emerged.

Our study found no substantial differences in AMR in *E. coli* between poultry production types. The finding that native poultry are also frequently contaminated with AMR organisms exhibiting resistance to the same antimicrobials used in commercial layers and broilers is remarkable, as native chickens are raised in a free-range systems (i.e., subsistence-based households) perceived not to be treated with any antimicrobials. A possible explanation is that these birds come in contact with (resistant *E. coli*- and *Salmonella* spp.-harboring) pigs, commercial poultry, and/or humans, or that they have been sourced from local breeders, hatcheries, or suppliers with historical or recent AMU exposure (via vertical spread from parents to progeny, or via horizontal spread from hatcheries).

The authors recognize that this pilot AMR surveillance study has several limitations. First, the model for the sample size was patterned from the EFSA, who stated that the optimal sample size should be 170 positive isolates of *E. coli* and *Salmonella* spp. in each animal species/breed. However, it was not possible for every breed, species, sampling period, and location (province) to collect 350 samples to harvest 170 *Salmonella* spp. isolates, which is arguably not a strong enough sample size to evaluate resistance in *Salmonella* spp. by different groups, locations, or times (Table 1). Secondly, it was assumed that the 10 samples from each slaughterhouse (pigs) and market (poultry) would represent animals from 10 different locations/farms; however, animals could not be traced back to their farm of origin, which could have resulted in biased sample collection (resistance data clustered in multiple samples from the same farm). Thirdly, AMR surveillance in pigs was conducted over two periods, but in different provinces in each period. Thus, no interpretation or explanation could be given to the apparent increasing spatial or temporal AMR trends for ciprofloxacin and nalidixic acid for this species. Fourthly, *Salmonella* spp. isolates have not been serotyped, hampering the interpretation of the resistance data, as resistance patterns may differ strongly for different serotypes. Finally, the data provided and used in this study were unsuitable for conducting multivariable analysis to identify possible risk factors, as was done by Tuat et al. (2021) [4]. Estimates were adjusted for province in our study to account for similarities in the production practices within the province, and possibly animals from the same farm/establishment (or located in close proximity) within the province, with subanalysis of the differences in breed (in poultry) and rounds of collection (in pigs), but further work is required in order to identify where clustering could have occurred (i.e., within sampling days, districts, slaughterhouses, or wet markets). With regard to the laboratory methods, the lack of interpretative criteria for the full panel of antimicrobials used in this study (i.e., based on EUCAST) using the MIC technique prompted the researchers to utilize available criteria (e.g., azithromycin, sulfamethoxazole, tetracycline, nalidixic acid), such as those of the CLSI, and harmonize them with other existing AMR surveillance programs [27,28]. For these antimicrobials, the MIC distributions (and median MIC values) obtained from the chicken and pig data were within the CLSI’s recommended concentration ranges, which the authors acknowledged to be appropriate for describing the data. However, further data collection could be added to these initial results to determine which CBPs to use that may be more suitable for the country/region.

These initial data could be used to refine regulations pertaining to veterinary medicines and stewardship interventions in the food animal sector. In developing countries, including the Lao PDR—and especially in the livestock sector—weak or non-existent regulatory frameworks on veterinary AMU, suboptimal enforcement and compliance with existing AMU guidelines, low levels of AMR awareness, poor farmer and poor veterinary education, and inadequate commitment to antimicrobial stewardship are some of the drivers of AMR. These issues were identified in a more recent study in the Lao PDR that mapped the veterinary antimicrobial distribution chain and analyzed the roles and interactions of key players [32]; the study cites the lack of veterinarian–farmer interaction and the evolving nature of the veterinary antimicrobial supply chain as factors that could impact changes in behavior with regard to AMU/AMR, through regulations amending veterinary AMU. In the same study, it was found that most of the antimicrobials found on farms are those categorized by the WHO as HPCIAs and CIAs [31]. From a public health perspective, it is especially important to monitor the dispensation and quantity of antimicrobials in both animals and humans. Furthermore, understanding the role of human drug pharmacies as potential sources of antimicrobials for use in food animals could identify gaps in AMU regulations. To mitigate the risks of AMR, we recommend that, initially, a longitudinal national AMR surveillance program for livestock be established in the Lao PDR, complemented by AMU surveillance. Regular, annual surveillance makes it possible to assess the impact of interventions, observe trends, and provide advice on additional regulations or implementations that will contribute to reducing the public health threat and AMR-associated burden of illness in the country. In the near future, a One Health, integrated surveillance system involving a multisector collaborative nature (human health, animal health, and the private sector) should be formed to generate data from different sources. Ideally, an updated NSP-AMR in the Lao PDR [15] should identify long-term funding to support these One Health surveillance activities and ensure sustainability of such an integrated longitudinal surveillance program. Our study serves as a reference point to detect the impact of the NSP-AMR, and the surveillance methods (sampling and laboratory) could inform the refinements and implementation of AMR surveillance activities; more importantly, quantitative MIC data were generated that could contribute to the national/regional AMR databases.

## Figures and Tables

**Table 1:** Collection of pig and chicken caecal specimens during two rounds of antimicrobial resistance surveillance in five provinces in the Lao PDR, 2018–2021.

Province	Pig (Round 1, 2018–2019)	Pig (Round 2, 2020–2021)	Poultry (Round 2, 2020–2021)			Total (by Province)
			Native	Broiler	Layer	
Louangprabang	0	262	164	0	91	**517**
Xiengkhouang	0	125	75	0	0	**200**
Vientiane Capital	160	0	0	255	510	**925**
Savannakhet	350	0	224	0	0	**574**
Champasak	262	0	362	574	224	**1422**
**Total (by species and breed):**	**772**	**387**	**825**	**829**	**825**	**3638**

**Table 2:** Antimicrobial resistance phenotypes in *Escherichia coli* from chickens (*n* = 1110) and pigs (*n* = 754) in the Lao PDR, 2018–2021, and comparison of the differences in percentages between pigs (referent species) and chickens.

Resistance Phenotypes	Chickens % Isolates	Pigs % Isolates	Pigs Compared to Chickens (Odds Ratios, 95% CIs, and *p*-Values)
No resistance ^1^	6%	<1%	OR 0.06 (0.01–0.38), *p* = 0.003
1 Antimicrobial class	13%	3%	OR: 0.220 (0.09–0.52), *p* = 0.001
2 Multiclass resistance	19%	9%	OR: 0.43 (0.22–0.83), *p* = 0.011
3 Multiclass resistance	**24%**	20%	OR: 0.79(065–0.98), *p* = 0.036
4 Multiclass resistance	21%	**31%**	OR: 1.68 (1.49–1.90), *p* < 0.0001
5 Multiclass resistance	12%	18%	OR 1.68 (1.18–2.42), *p* = 0.004
6 Multiclass resistance	4%	10%	OR 2.4 2 (1.62–3.63), *p* < 0.0001
7 Multiclass resistance	2%	7%	OR 4.56 (2.54–8.29), *p* < 0.0001
8 Multiclass resistance	0.1%	2%	OR 22.51 (2.70–187.45), *p* = 0.004
≥3 Multiclass resistance ^2^	62%	88%	OR 4.29 (1.82–10.12), *p* = 0.001

^1^ Isolates that were not resistant to any of the antimicrobials included in the EUVSEC panel. ^2^ Isolates that exhibited resistance to antimicrobials belonging to 3 or more classes (aggregate of 3–7 multiclass categories). Percentages in bold represent the most frequently occurring multiclass resistance phenotype for the animal species. CI: confidence interval.

**Table 3:** Antimicrobial resistance phenotypes in *Salmonella* spp. from chickens (*n* = 698) and pigs (*n* = 675) in the Lao PDR, 2018–2021, and comparison of the differences in percentages between pigs (referent species) and chickens.

Resistance Phenotypes	Chickens	Pigs	Pigs Compared to Chickens (Odds Ratios, 95% CIs, and *p*-Values)
No resistance ^1^	15%	3%	OR 0.18 (0.07–0.46), *p* < 0.0001
1 Antimicrobial class	25%	7%	OR: 0.20 (0.10–0.46), *p* < 0.0001
2 Multiclass resistance	**27%**	10%	OR: 0.29 (0.16–0.51), *p* < 0.0001
3 Multiclass resistance	17%	**36%**	OR: 2.70 (1.86–3.93), *p* < 0.0001
4 Multiclass resistance	7%	16%	OR: 2.38 (1.72–3.29), *p* < 0.0001
5 Multiclass resistance	5%	18%	OR 3.78 (1.52–12.35), *p* = 0.028
6 Multiclass resistance	1%	6%	OR 1.58 (2.73–9.34), *p* < 0.0001
7 Multiclass resistance	1%	4%	OR 5.3 (1.10–25.44), *p* = 0.037
8 Multiclass resistance	<1%	<1%	OR 1.03 (0.12–8.26), *p* = 0.979
≥3 Multiclass resistance ^2^	33%	81%	OR 8.53 (4.1–17.5), *p* < 0.0001

^1^ Isolates that were not resistant to any of the antimicrobials included in the EUVSEC panel. ^2^ Isolates that exhibited resistance to antimicrobials belonging to 3 or more different classes (aggregate of 3–7 multiclass categories). Percentages in bold depict the most frequently occurring multiclass resistance phenotype for the animal species. CI: confidence interval.

## Data Availability

Not applicable.

## Supplementary Materials

The following are available online at https://www.mdpi.com/article/10.3390/antibiotics11020177/s1: Figure S1: Percentage of resistance in *Escherichia coli*, comparison between chicken breeds/production types (broilers, *n* = 373; layers, *n* = 346; and native chickens, *n* = 393) in the Lao PDR, 2018–2021. For each antimicrobial, the percentage of resistance was adjusted for clustering at the province level. Asterisks represent significant differences (*p* ≤ 0.05) for the given antimicrobial between chicken breeds/production types (layers—referent breed); Figure S2: Percentage of resistance in *Escherichia coli*, comparison between sampling rounds/provinces in pigs (round 1/2018–2019, *n* = 583 vs. round 2/2018–2019, *n* = 171) in the Lao PDR, 2018–2021. For each antimicrobial, the percentage of resistance was adjusted for clustering at the province level. Asterisks represent significant differences (*p* ≤ 0.05) for the given antimicrobial between rounds; Figure S3: Percentage of resistance in *Salmonella*, comparison between chicken breeds/production types (broilers, *n* = 268; layers, *n* = 257; and native chickens, *n* = 167) in the Lao PDR, 2018–2021. For each antimicrobial, the percentage of resistance was adjusted for clustering at the province level. Asterisks represent significant differences (*p* ≤ 0.05) for the given antimicrobial between chicken breeds/production types (layers—referent breed); Figure S4: Percentage of resistance in *Salmonella*, comparison between sampling rounds/provinces in pigs (round 1/2018–2019, *n* = 411 vs. round 2/2018–2019, *n* = 262) in the Lao PDR, 2018–2021. For each antimicrobial, the percentage of resistance was adjusted for clustering at the province level. Asterisks represent significant differences (*p* ≤ 0.05) for the given antimicrobial between rounds; Table S1: Minimum inhibitory concentration (MIC) interpretative criteria used in this study; Table S2: Antimicrobial resistance patterns in multiclass-resistant *Escherichia coli* isolates; Table S3: Antimicrobial resistance patterns in multiclass-resistant *Salmonella* spp. isolates.
