# Supplementary material for: Antimicrobial Resistance Surveillance of Pigs and Chickens in the Lao People’s Democratic Republic, 2018–2021
Source: Antibiotics (Basel). 2022 Jan 29;11(2):177. doi: 10.3390/antibiotics11020177 (PMC8868105; doi:10.3390/antibiotics11020177)
Supplement: Supplementary file 1 [file antibiotics-11-00177-s001.zip › antibiotics-1526053-supplementary.pdf]

Supplementary Materials

Figure S1. Percentage of resistance in *Escherichia coli*, comparison between chicken breeds/production types (broilers, n = 373, layers, n = 346 and native chickens, n = 393) in Lao PDR, 2018-2021. For each antimicrobial, the percentage of resistance was adjusted for clustering at the province level. Asterisks represent significant difference ($p \leq 0.05$), for that antimicrobial, between chicken breeds/production types (layers-referent breed).

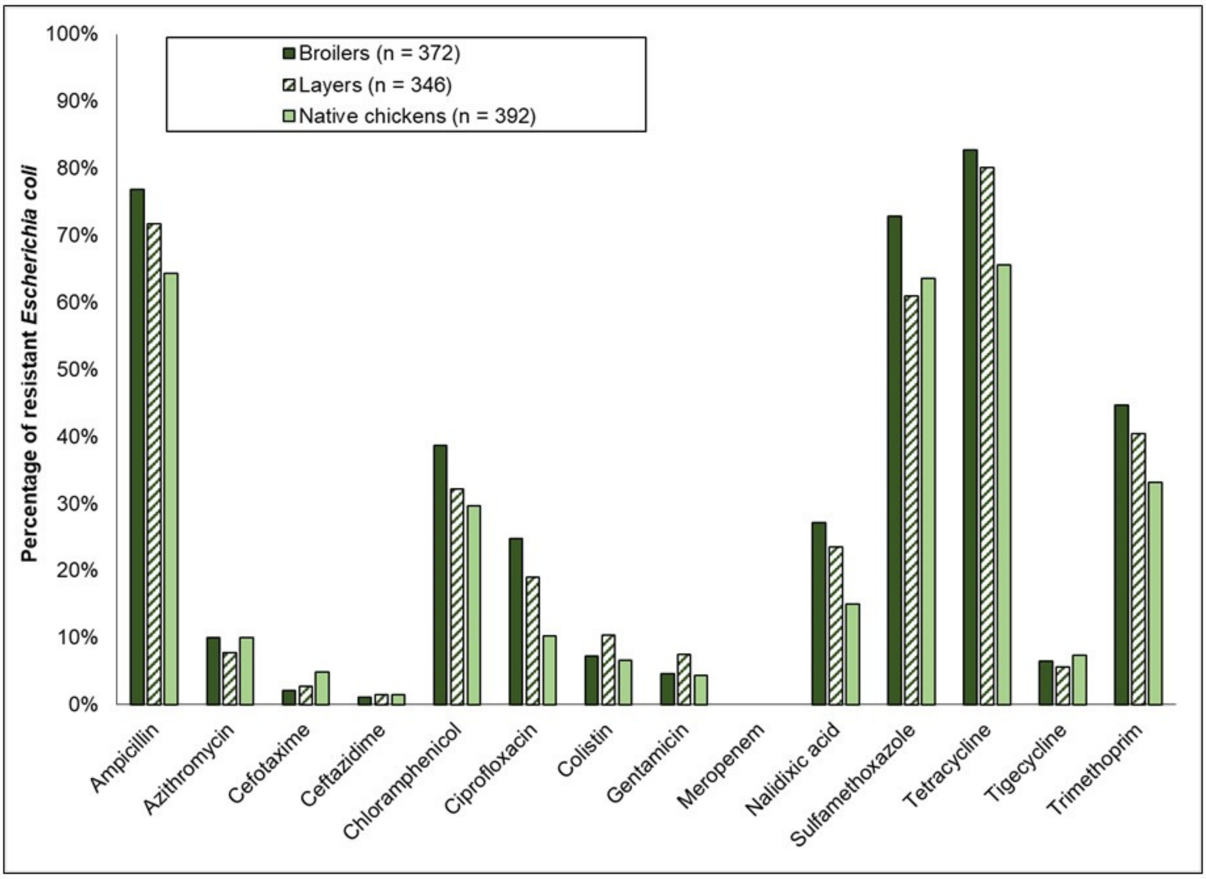

Figure S2. Percentage of resistance in *Escherichia coli*, comparison between sampling rounds/provinces in pigs, (Round 1/2018-2019, n = 583) vs. (Round 2/2018-2019, n = 171) in Lao PDR, 2018-2021. For each antimicrobial, the percentage of resistance was adjusted for clustering at the province level. Asterisks represent significant difference ($p \leq 0.05$), for that antimicrobial, between round.

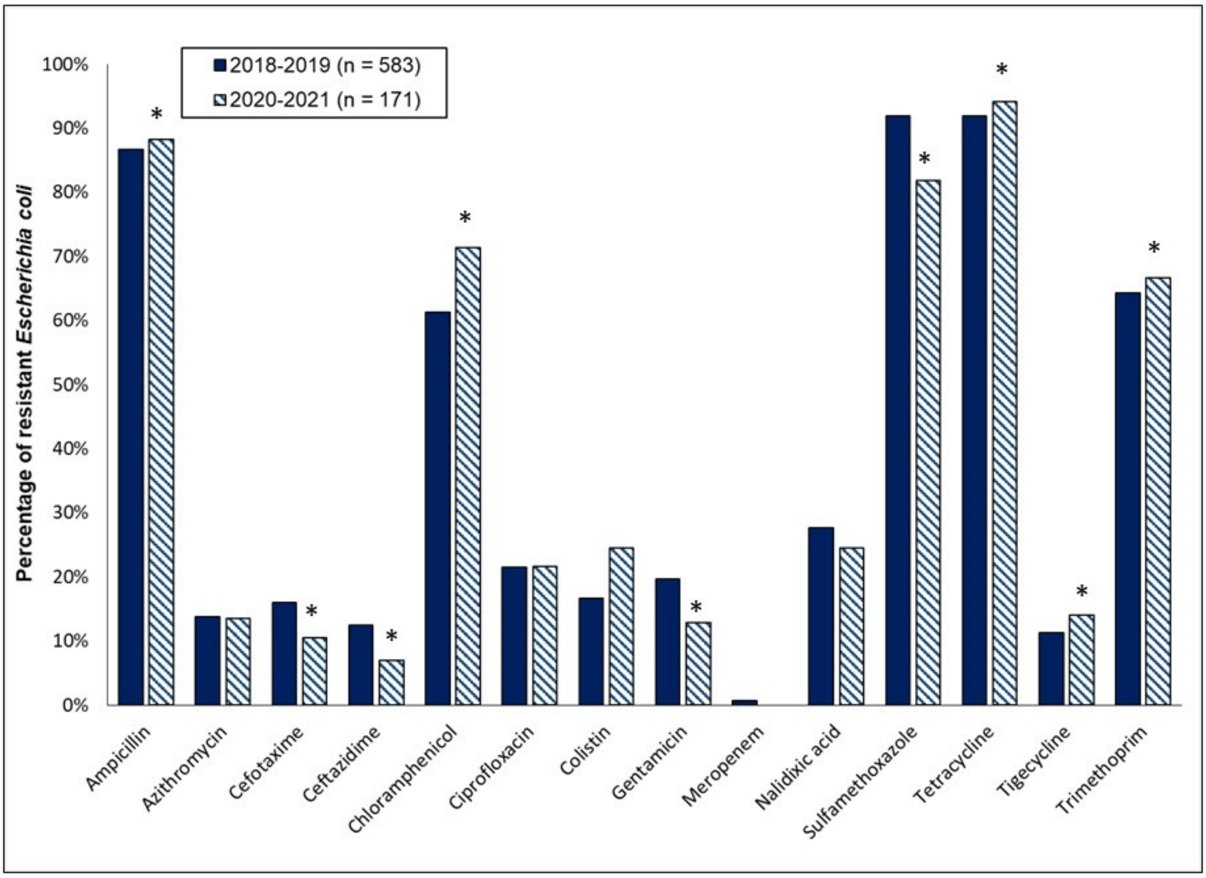

Figure S3. Percentage of resistance in *Salmonella*, comparison between chicken breeds/production types (broilers, n = 268, layers, n = 257 and native chickens, n = 167) in Lao PDR, 2018-2021. For each antimicrobial, the percentage of resistance was adjusted for clustering at the province level. Asterisks represent significant difference ($p \leq 0.05$), for that antimicrobial, between chicken breeds/production types (layers-referent breed).

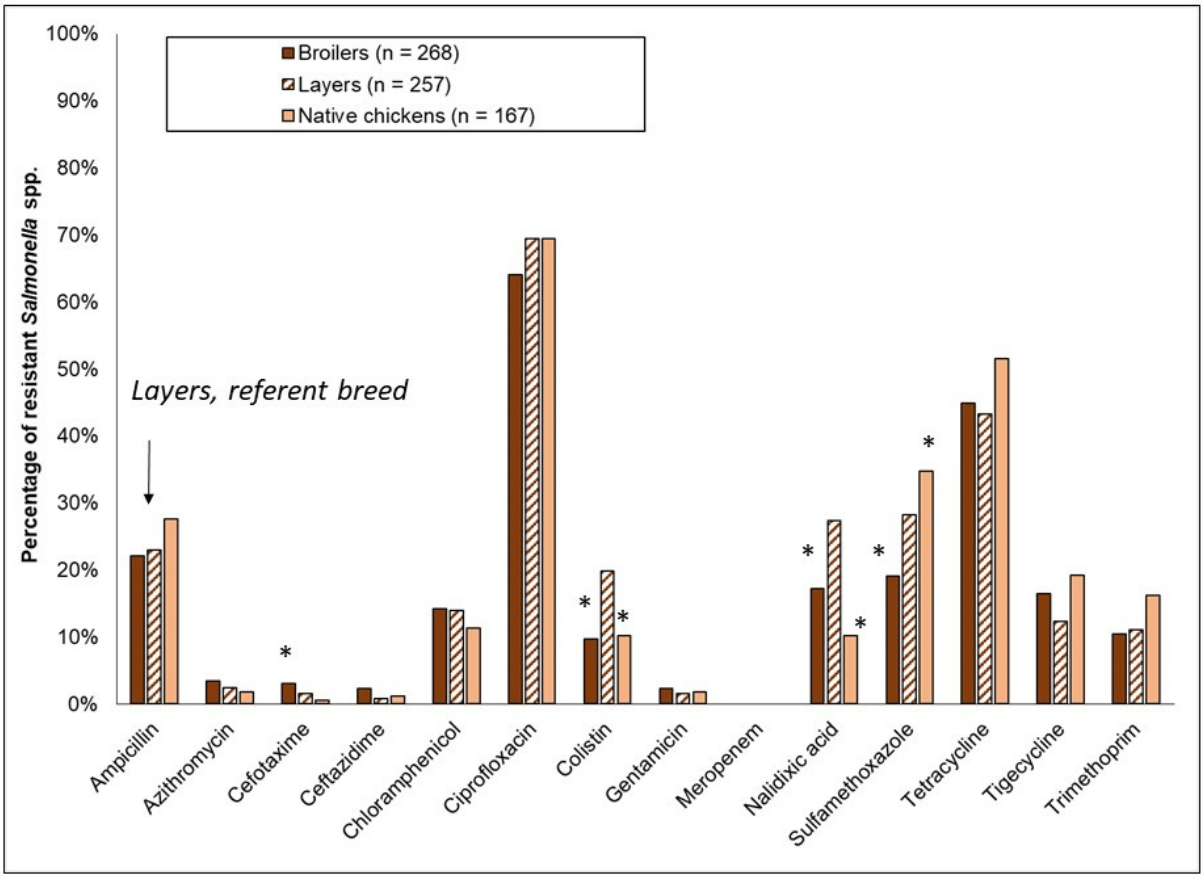

Figure S4. Percentage of resistance in *Salmonella,* comparison between sampling rounds/provinces in pigs, (Round 1/2018-2019, n = 411) vs. (Round 2/2018-2019, n = 262) in Lao PDR, 2018-2021. For each antimicrobial, the percentage of resistance was adjusted for clustering at the province level. Asterisks represent significant difference ($p \leq 0.05$), for that antimicrobial, between round.

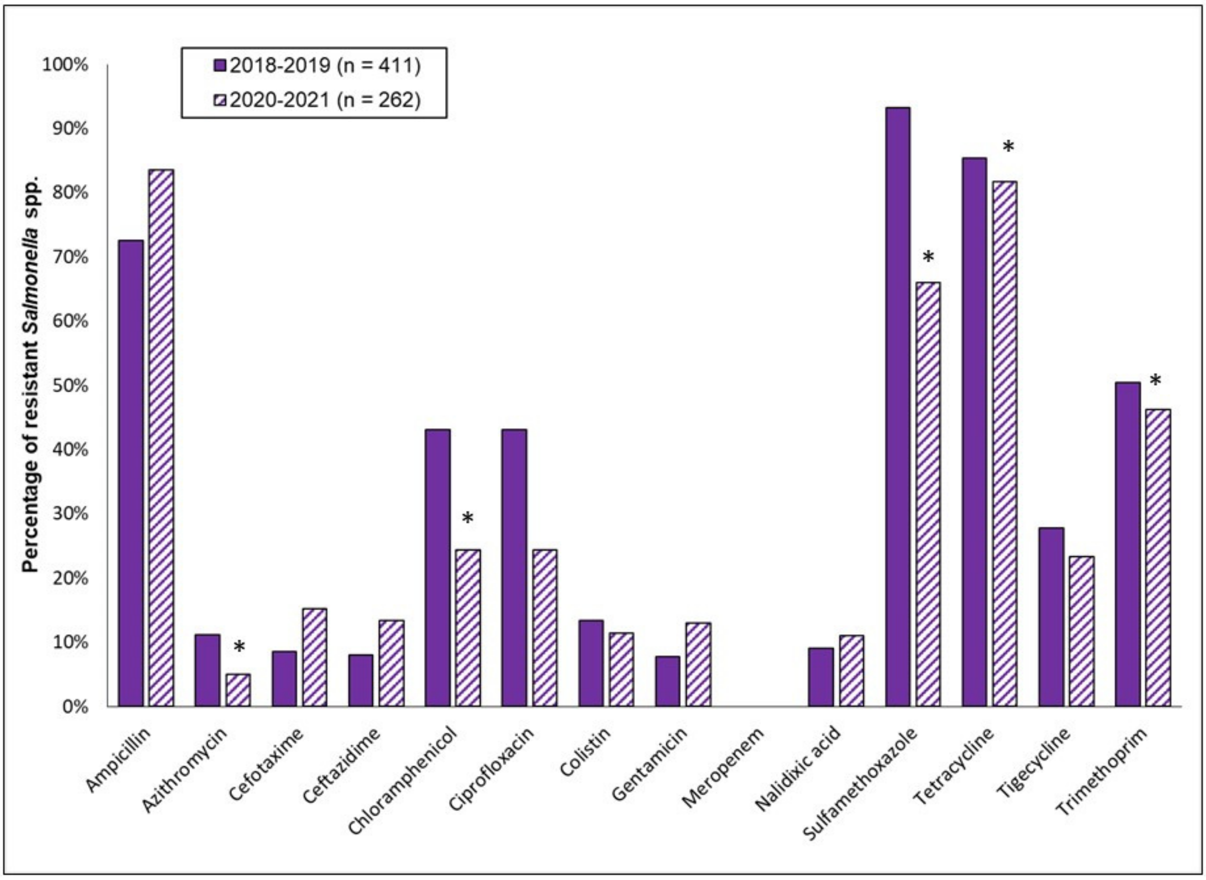

Table S1. Minimum inhibitory concentration (MIC) interpretative criteria used in this study.

	MIC Interpretative criteria used	Clinical breakpoints *E. coli*	Clinical breakpoints *Salmonella*
Ampicillin	EUCAST[1]	> 8	> 8
Azithromycin	CLSI[2]	≥ 32	≥ 32
Cefotaxime	EUCAST	>2	>2
Ceftazidime	EUCAST	>4	>4
Chloramphenicol	EUCAST	>8	>8
Ciprofloxacin	EUCAST	>0.5	>0.06
Colistin	EUCAST	>2	>2
Gentamicin	EUCAST	>2	>2
Meropenem	EUCAST	>8	>8
Nalidixic acid	CLSI	≥32	≥32
Sulfamethoxazole	CLSI	≥512	≥512
Tetracycline	CLSI	≥16	≥16
Tigecycline	EUCAST	>0.5	>0.5
Trimethoprim	EUCAST	>4	>4

[1] EUCAST. The European Committee on Antimicrobial Susceptibility Testing - EUCAST (www.EUCAST.org).

[2] CLSI, Clinical Laboratory Standard Institute [26] and harmonized with other national AMR surveillance systems [27,28]; interim breakpoints used in the absence of EUCAST breakpoints

Please note: *Escherichia coli* ATCC 25922 was the reference strain used in the study which is the bacterial strain for quality control indicated in both interpretative criteria.

Table S2. Antimicrobial resistance patterns in multiclass resistant *Escherichia coli* isolates

KEY:
amin – aminoglycosides; bla – beta lactam antimicrobials (3rd generation cephalosporins, aminopenicillins and cabapenems); fol – folate pathway inhibitors; mac – macrolides; phe – phenicols, poly – polymyxins; qnl – quinolones/fluoroquinolones; tetr – tetracyclines,

A. Chickens (n =1039 antimicrobial class patterns)

Antimicrobial resistance patterns	Number of isolates in the pattern	Percent
bla-fol-tetr-	155	14.92
bla-fol-phe-tetr-	131	12.61
bla-tetr-	104	10.01
bla-fol-qnl-phe-tetr-	74	7.12
fol-	70	6.74
fol-tetr-	48	4.62
bla-fol-qnl-tetr-	46	4.43
tetr-	33	3.18
bla-fol-	28	2.69
fol-phe-tetr-	28	2.69
bla-	25	2.41
bla-fol-mac-tetr-	19	1.83
bla-fol-mac-qnl-phe-tetr-	17	1.64
bla-fol-phe-poly-tetr-	17	1.64
bla-fol-mac-phe-tetr-	15	1.44
amn-bla-fol-qnl-phe-tetr-	13	1.25
bla-mac-tetr-	13	1.25
fol-qnl-tetr-	13	1.25
bla-qnl-tetr-	12	1.15
bla-fol-qnl-phe-poly-tetr-	10	0.96
bla-fol-poly-tetr-	9	0.87
bla-fol-qnl-	8	0.77
bla-poly-tetr-	8	0.77
amn-bla-fol-qnl-phe-poly-tetr-	7	0.67
bla-fol-mac-qnl-tetr-	7	0.67
bla-phe-tetr-	7	0.67
amn-bla-fol-mac-qnl-phe-tetr-	6	0.58
amn-bla-fol-qnl-tetr-	6	0.58
bla-fol-phe-	6	0.58
qnl-	6	0.58
qnl-tetr-	6	0.58
amn-bla-fol-phe-tetr-	5	0.48
bla-fol-mac-qnl-phe-poly-tetr-	5	0.48
amn-bla-fol-tetr-	4	0.38
amn-fol-	4	0.38

phe-	4	0.38
poly-tetr-	4	0.38
amn-bla-fol-mac-phe-tetr-	3	0.29
bla-fol-mac-poly-tetr-	3	0.29
bla-fol-qnl-phe-	3	0.29
fol-phe-	3	0.29
fol-qnl-	3	0.29
fol-qnl-phe-	3	0.29
fol-qnl-phe-tetr-	3	0.29
fol-qnl-poly-tetr-	3	0.29
poly-	3	0.29
amn-bla-fol-qnl-	2	0.19
amn-bla-tetr-	2	0.19
bla-fol-mac-	2	0.19
bla-fol-mac-phe-poly-tetr-	2	0.19
bla-fol-phe-poly-	2	0.19
bla-qnl-	2	0.19
fol-mac-qnl-tetr-	2	0.19
fol-poly-	2	0.19
amn-bla-fol-mac-qnl-phe-poly-tetr-	1	0.10
amn-bla-fol-mac-qnl-tetr-	1	0.10
amn-bla-fol-poly-tetr-	1	0.10
amn-bla-fol-qnl-poly-tetr-	1	0.10
amn-bla-qnl-	1	0.10
amn-fol-qnl-phe-tetr-	1	0.10
amn-qnl-poly-	1	0.10
bla-fol-mac-phe-poly-	1	0.10
bla-fol-qnl-poly-	1	0.10
bla-mac-phe-tetr-	1	0.10
bla-mac-poly-tetr-	1	0.10
bla-mac-qnl-phe-tetr-	1	0.10
bla-mac-qnl-tetr-	1	0.10
bla-qnl-poly-	1	0.10
bla-qnl-poly-tetr-	1	0.10
fol-mac-tetr-	1	0.10
fol-phe-poly-tetr-	1	0.10
fol-poly-tetr-	1	0.10
fol-qnl-poly-	1	0.10
phe-tetr-	1	0.10
qnl-phe-tetr-	1	0.10
qnl-poly-	1	0.10
qnl-poly-tetr-	1	0.10

B. Pigs (n = 751 antimicrobial class patterns)

Antimicrobial resistance patterns	Number of isolates in the pattern	Percent
bla-fol-phe-tetr-	174	23.17
bla-fol-tetr-	124	16.51
bla-fol-qnl-phe-tetr-	57	7.59
fol-tetr-	33	4.39
amn-bla-fol-phe-tetr-	32	4.26
bla-fol-phe-poly-tetr-	25	3.33
bla-tetr-	23	3.06
bla-fol-mac-qnl-phe-poly-tetr-	22	2.93
amn-bla-fol-mac-qnl-phe-tetr-	18	2.40
bla-fol-qnl-tetr-	18	2.40
amn-bla-fol-qnl-phe-tetr-	16	2.13
amn-bla-fol-mac-qnl-phe-poly-tetr-	15	2.00
bla-fol-qnl-phe-poly-tetr-	15	2.00
amn-bla-fol-phe-poly-tetr-	14	1.86
fol-	14	1.86
amn-bla-fol-qnl-phe-poly-tetr-	12	1.60
bla-fol-mac-qnl-phe-tetr-	10	1.33
fol-phe-tetr-	10	1.33
bla-fol-	8	1.07
bla-fol-qnl-poly-tetr-	8	1.07
bla-fol-mac-phe-poly-tetr-	7	0.93
bla-fol-phe-	6	0.80
fol-qnl-phe-tetr-	6	0.80
amn-bla-fol-mac-phe-tetr-	5	0.67
amn-bla-fol-tetr-	5	0.67
bla-fol-mac-phe-tetr-	5	0.67
bla-fol-poly-tetr-	5	0.67
tetr-	5	0.67
amn-fol-phe-tetr-	4	0.53
bla-fol-qnl-phe-	4	0.53
amn-bla-fol-phe-	3	0.40
bla-	3	0.40
bla-fol-mac-qnl-poly-tetr-	3	0.40
bla-fol-mac-qnl-tetr-	3	0.40
bla-phe-tetr-	3	0.40
amn-bla-fol-mac-qnl-phe-	2	0.27
amn-bla-fol-poly-tetr-	2	0.27
bla-fol-mac-tetr-	2	0.27
bla-qnl-phe-tetr-	2	0.27
amn-bla-fol-mac-phe-	1	0.13
amn-bla-fol-mac-phe-poly-tetr-	1	0.13
amn-bla-fol-mac-tetr-	1	0.13

amn-bla-fol-qnl-	1	0.13
amn-bla-fol-qnl-tetr-	1	0.13
amn-bla-tetr-	1	0.13
amn-fol-qnl-phe-tetr-	1	0.13
amn-fol-tetr-	1	0.13
amn-qnl-poly-tetr-	1	0.13
bla-fol-mac-	1	0.13
bla-fol-mac-qnl-phe-poly-	1	0.13
bla-fol-mac-qnl-poly-	1	0.13
bla-fol-phe-poly-	1	0.13
bla-fol-qnl-	1	0.13
bla-fol-qnl-phe-poly-	1	0.13
bla-mac-phe-tetr-	1	0.13
bla-mac-poly-tetr-	1	0.13
bla-mac-tetr-	1	0.13
bla-phe-	1	0.13
bla-qnl-tetr-	1	0.13
fol-mac-phe-tetr-	1	0.13
fol-mac-qnl-phe-tetr-	1	0.13
fol-phe-poly-	1	0.13
fol-phe-poly-tetr-	1	0.13
fol-qnl-	1	0.13
fol-qnl-tetr-	1	0.13
poly-	1	0.13
poly-tetr-	1	0.13

Table S3. Antimicrobial resistance patterns in multiclass resistant *Salmonella* spp. isolates

A. Chickens (n = 698 antimicrobial class patterns)

Multiclass resistance patterns	Number of isolates in the pattern	Percent
qnl-	120	20.13
fol-qnl-tetr-	52	8.72
qnl-poly-	37	6.21
bla-fol-qnl-phe-tetr-	29	4.87
bla-fol-qnl-tetr-	26	4.36
fol-	18	3.02
bla-fol-tetr-	15	2.52
bla-qnl-tetr-	14	2.35
fol-qnl-	14	2.35
bla-tetr-	13	2.18
qnl-poly-tetr-	11	1.85
fol-tetr-	10	1.68
qnl-phe-	10	1.68
bla-fol-qnl-	8	1.34
bla-qnl-	7	1.17
poly-	6	1.01
qnl-phe-tetr-	6	1.01
bla-qnl-poly-	5	0.84
fol-qnl-phe-tetr-	5	0.84
bla-fol-mac-qnl-phe-tetr-	4	0.67
bla-fol-qnl-poly-tetr-	4	0.67
bla-fol-phe-tetr-	3	0.5
bla-fol-poly-tetr-	3	0.5
bla-poly-	3	0.5
bla-qnl-phe-tetr-	3	0.5
amn-bla-fol-mac-qnl-phe-poly-tetr-	2	0.34
amn-bla-fol-mac-qnl-phe-tetr-	2	0.34
amn-bla-fol-qnl-phe-poly-tetr-	2	0.34
amn-bla-phe-tetr-	2	0.34
bla-	2	0.34
bla-fol-qnl-phe-	2	0.34
bla-fol-qnl-phe-poly-tetr-	2	0.34
bla-qnl-phe-	2	0.34
bla-qnl-poly-tetr-	2	0.34
fol-qnl-phe-	2	0.34
fol-qnl-poly-	2	0.34
fol-qnl-poly-tetr-	2	0.34
phe-	2	0.34
phe-tetr-	2	0.34
amn-bla-fol-phe-tetr-	1	0.17
amn-bla-fol-qnl-phe-tetr-	1	0.17
amn-fol-poly-	1	0.17
amn-phe-	1	0.17

amn-qnl-	1	0.17
bla-fol-	1	0.17
bla-fol-mac-phe-tetr-	1	0.17
bla-fol-mac-poly-tetr-	1	0.17
bla-fol-mac-qnl-phe-	1	0.17
bla-fol-mac-qnl-phe-poly-	1	0.17
bla-fol-mac-qnl-phe-poly-tetr-	1	0.17
bla-fol-mac-qnl-poly-tetr-	1	0.17
bla-fol-mac-tetr-	1	0.17
bla-fol-phe-	1	0.17
bla-mac-qnl-poly-	1	0.17
bla-mac-qnl-poly-tetr-	1	0.17
bla-phe-	1	0.17
bla-poly-tetr-	1	0.17
fol-phe-poly-tetr-	1	0.17
fol-poly-	1	0.17
fol-qnl-phe-poly-tetr-	1	0.17
mac-qnl-	1	0.17
phe-poly-	1	0.17
poly-tetr-	1	0.17
qnl-phe-poly-	1	0.17

B. Pigs (n = 675 antimicrobial class patterns)

Multiclass resistance patterns	Number of isolates in the pattern	Percent
bla-fol-tetr-	174	26.65
bla-fol-qnl-phe-tetr-	78	11.94
bla-fol-phe-tetr-	71	10.87
fol-qnl-tetr-	47	7.2
fol-	33	5.05
bla-tetr-	32	4.9
amn-bla-fol-qnl-phe-tetr-	14	2.14
bla-fol-	13	1.99
bla-fol-qnl-phe-poly-tetr-	13	1.99
fol-tetr-	12	1.84
amn-bla-fol-qnl-phe-poly-tetr-	10	1.53
amn-bla-phe-tetr-	10	1.53
bla-fol-qnl-tetr-	10	1.53
bla-fol-mac-qnl-tetr-	9	1.38
amn-bla-fol-mac-qnl-phe-tetr-	8	1.23
amn-bla-fol-phe-tetr-	7	1.07
bla-fol-mac-qnl-phe-poly-tetr-	6	0.92
bla-fol-phe-poly-tetr-	6	0.92
bla-fol-poly-tetr-	6	0.92
bla-phe-tetr-	6	0.92
bla-	5	0.77

amn-bla-fol-mac-tetr-	4	0.61
bla-fol-mac-qnl-phe-tetr-	4	0.61
bla-fol-mac-qnl-poly-tetr-	4	0.61
bla-fol-qnl-poly-tetr-	4	0.61
qnl-	4	0.61
qnl-poly-	4	0.61
bla-fol-mac-poly-tetr-	3	0.46
bla-fol-qnl-	3	0.46
fol-phe-tetr-	3	0.46
fol-poly-	3	0.46
fol-qnl-phe-tetr-	3	0.46
amn-bla-fol-mac-qnl-phe-poly-tetr-	2	0.31
bla-fol-mac-phe-tetr-	2	0.31
bla-fol-phe-	2	0.31
bla-mac-qnl-phe-poly-tetr-	2	0.31
fol-mac-phe-poly-tetr-	2	0.31
fol-mac-poly-tetr-	2	0.31
fol-poly-tetr-	2	0.31
qnl-poly-tetr-	2	0.31
qnl-tetr-	2	0.31
tetr-	2	0.31
amn-bla-fol-phe-poly-tetr-	1	0.15
amn-bla-fol-poly-tetr-	1	0.15
amn-bla-fol-qnl-poly-tetr-	1	0.15
amn-bla-fol-qnl-tetr-	1	0.15
amn-bla-mac-phe-tetr-	1	0.15
amn-bla-phe-poly-tetr-	1	0.15
amn-bla-qnl-poly-	1	0.15
amn-fol-mac-phe-poly-tetr-	1	0.15
amn-fol-mac-poly-tetr-	1	0.15
amn-fol-mac-qnl-phe-poly-tetr-	1	0.15
amn-fol-mac-qnl-phe-tetr-	1	0.15
bla-fol-mac-phe-poly-tetr-	1	0.15
bla-fol-mac-qnl-	1	0.15
bla-fol-poly-	1	0.15
bla-mac-poly-tetr-	1	0.15
bla-mac-qnl-tetr-	1	0.15
bla-qnl-phe-	1	0.15
bla-qnl-poly-	1	0.15
bla-qnl-tetr-	1	0.15
fol-mac-qnl-poly-tetr-	1	0.15
fol-qnl-poly-tetr-	1	0.15
mac-qnl-tetr-	1	0.15
qnl-phe-	1	0.15
qnl-phe-tetr-	1	0.15
